# A Novel Hemp Seed Meal Protein Hydrolysate Reduces Oxidative Stress Factors in Spontaneously Hypertensive Rats

**DOI:** 10.3390/nu6125652

**Published:** 2014-12-08

**Authors:** Abraham T. Girgih, Adeola M. Alashi, Rong He, Sunday A. Malomo, Pema Raj, Thomas Netticadan, Rotimi E. Aluko

**Affiliations:** 1 Department of Human Nutritional Sciences and the Richardson Centre for Functional Foods and Nutraceuticals, University of Manitoba, Winnipeg, MB R3T 2N2, Canada; E-Mails: umgirgia@myumanitoba.ca (A.T.G.); aalashi@csu.edu.au (A.M.A.); rong.he@njue.edu.cn (R.H.); malomos@myumanitoba.ca (S.A.M.); 2 School of Agriculture and Wine Sciences, Charles Sturt University, Bag 588, Wagga Wagga, NSW 2678, Australia; 3 College of Food Science and Engineering, Nanjing University of Finance and Economics, Nanjing 210046, China; 4 Department of Physiology, University of Manitoba, Winnipeg, MB R3T 2N2, Canada; E-Mails: pemaraj@gmail.com (P.R.); tnetticadan@sbrc.ca (T.N.); 5 Canadian Centre for Agri-Food Research in Health and Medicine, 351 Tache Avenue, Winnipeg, MB R2H 2A6, Canada

**Keywords:** hemp seed, antioxidant peptides, protein hydrolysate, spontaneously hypertensive rats, total antioxidant capacity, pepsin, pancreatin, defatted meal

## Abstract

This report shows the antioxidant effects of a hemp seed meal protein hydrolysate (HMH) in spontaneously hypertensive rats (SHR). Defatted hemp seed meal was hydrolyzed consecutively with pepsin and pancreatin to yield HMH, which was incorporated into rat feed as a source of antioxidant peptides. Young (8-week old) SHRs were divided into three groups (8 rats/group) and fed diets that contained 0.0%, 0.5% or 1.0% (w/w) HMH for eight weeks; half of the rats were sacrificed for blood collection. After a 4-week washout period, the remaining 20-week old SHRs were fed for an additional four weeks and sacrificed for blood collection. Plasma total antioxidant capacity (TAC) and superoxide dismutase (SOD), catalase (CAT) and total peroxides (TPx) levels were determined. Results showed that plasma TAC, CAT and SOD levels decreased in the older 20-week old SHRs when compared to the young SHRs. The presence of HMH in the diets led to significant (*p* < 0.05) increases in plasma SOD and CAT levels in both young and adult SHR groups; these increases were accompanied by decreases in TPx levels. The results suggest that HMH contained antioxidant peptides that reduced the rate of lipid peroxidation in SHRs with enhanced antioxidant enzyme levels and total antioxidant capacity.

## 1. Introduction

*Cannabis sativa* L., also commonly called industrial hemp seed, is historically an important source of food, fibre, dietary oil and medicine; the seed contains about 30% oil and 25% protein [1]. Hemp seed storage proteins consist mainly of globulin (edestin) and albumin [2], which have higher digestibility (88%–91%) when compared to soy protein (71%) after hydrolysis with pepsin and trypsin enzymes [3]. There have been several claims of the health benefits of hemp seed proteins, and research recently has focused on validating these claims through coordinated laboratory tests and animal studies. The effect of limited or extensive enzymatic protein hydrolysis as a means of improving the functional properties of hemp seed proteins have been reported by different researchers [2,4]. Proteins from both plant and animal sources, including those of hemp seed, have been isolated and recognized as essential sources of bioactive peptides capable of exerting various *in vitro* and *in vivo* activities, such as antioxidant, antihypertensive, antimicrobial, opioid, antithrombotic, hypocholesterolemic, appetite-reducing, mineral-binding, immunomodulatory and cytomodulatory [5,6,7]. Extensive research works have been done on the *in vitro* antioxidant properties of hemp seed peptides with evidence of the ability to scavenge toxic free radicals, chelate metal ions and inhibit linoleic acid oxidation [8,9].

It is well understood that cell survival and normal physiological functions of a healthy body are highly dependent on oxidative metabolism. However, a persistent stressful or disease condition triggers the over production of free radicals and reactive oxygen species (ROS) that can cause deleterious oxidative changes. The formation of excessive free radicals and ROS in the body could readily overwhelm the normal protective endogenous antioxidative enzymes. A high oxidative stress condition can cause destructive and lethal cellular effects (e.g., apoptosis, inflammation) through extensive oxidation of critical biopolymers, such as DNA, membrane lipids, structural proteins and enzymes; thus, normal cellular processes can be shut down [7]. To downregulate oxidative stress, the organism possesses several biological defense mechanisms that involve activities of enzymes, such as superoxide dismutase (SOD), catalase (CAT) and glutathione peroxidase (GPx), as well as non-enzymatic antioxidants, such as carotenoids, vitamin C and glutathione [10].

Food-derived antioxidant peptides that may contain 2–20 amino acid residues are considered natural and potentially safe antioxidant resources in comparison to synthetic compounds, such as butylated hydroxyanisole (BHA) and butylated hydroxytoluene (BHT) [11]. BHA, BHT and propyl gallate are added to food products to retard lipid oxidation, a major cause of food quality deterioration that results in the development of rancidity and off-flavours [12]. However, the use of synthetic antioxidants has been limited due to health and safety concerns. Antioxidant peptides derived from natural sources have potential health benefits that are associated with low molecular weight, low cost, high activity, easy absorption and little or no negative side effects [11]. Previous works have shown the production of novel peptide antioxidants from animal sources through enzymatic hydrolysis of blood plasma, shrimp, milk and egg white proteins [13,14,15,16]. Antioxidant peptides have also been produced through enzymatic hydrolysis of plant food proteins from sunflower, sweet potato, flaxseed and chick pea [17,18,19,20]. *In vitro* experiments have also shown that peptides produced from enzymatic hydrolysis of hemp seed proteins possess antioxidant properties [8,9,21], but there is scant information on *in vivo* activity*.* Therefore, the objective of this study was to determine, for the first time, the ability of an antioxidant hemp seed meal protein hydrolysate (HMH) to attenuate plasma levels of some oxidative stress markers (antioxidant enzymes and lipid peroxides) in young (growing) and adult spontaneously hypertensive rats (SHRs). The SHR represents an excellent model of oxidative stress, since the vascular system has been shown to exhibit excessive levels of superoxide radicals that lead to hypertension in the adult rats [22]. Moreover, it was shown that administration of an antioxidant (Tempol) to young SHR prevented oxidative stress development in the adult rats [22]. Wistar Kyoto (WKY) rats were used as a control for the animal disease model.

## 2. Experimental Section

### 2.1. Materials

Hemp seed protein meal (25% protein content) produced as a by-product of the hemp seed oil processing industry was a gift from Hemp Oil Canada (St. Agathe, MB, Canada). 2,2-diphenyl-l-picrylhydrazyl (DPPH), Triton X-100, pyrogallol, ethylenediaminetetraacetic acid (EDTA), hydrogen peroxide, 1,10-phenanthroline, ferrous sulfate, linoleic acid, ammonia thiocyanate and ferrous chloride were purchased from Sigma-Aldrich (St. Louis, MO, USA), while other analytical-grade reagents were obtained from Fisher Scientific (Oakville, ON, Canada). Hemp seed protein isolate (HPI) and HMH were produced according to previously described methods and contained ~99% and 64% protein contents (dry weight basis), respectively [23]. Amino acid composition data showed that the main differences between HMH and HPI were the tryptophan (12.56% and 1.39%, respectively) and arginine (2.11% and 13.91%, respectively) contents [23].

### 2.2. In Vitro Antioxidant Assays

The radical scavenging activity of the HMH was first determined using standard *in vitro* tests for DPPH [24], hydroxyl [25] and superoxide [26] and compared with glutathione (GSH) as the positive standard. The metal (iron) chelating activity (MCA) was measured using a modified method of Xie *et al.* [27]. Peptide or GSH sample solution (final assay concentration of 1 mg/mL) was combined with 0.05 mL of 2 mM FeCl_2_ and 1.85 mL double-distilled water in a reaction test tube. Ferrozine solution (0.1 mL of 5 mM) was added and mixed thoroughly. The mixture was then allowed to stand at room temperature for 10 min, from which an aliquot of 200 μL was removed and added to a clear bottom 96-well plate. A blank was also conducted by replacing the sample with 1 mL of double-distilled water. The absorbance values of the blank (A_b_) and sample (A_s_) at 562 nm were measured using a spectrophotometer. The percentage of the chelating effect (%) was calculated using the following equation.

MCA (%) = ((A_b_ − A_s_) × 100)/A_b_ (1)

### 2.3. Animal Studies

All rat experiments were performed according to protocols approved by the University of Manitoba Animal Care Protocol and Management Review Committee. The rat feeding experiments were carried out as follows using SHR (hypertensive) or normotensive WKY rats (NTR) purchased from Charles River Laboratories (Montreal, PQ, Canada). Details of the chemical composition of HPI and HMH, as well as the ingredient composition of rat diets and feed groups have been previously described [23]. Briefly, 32 6-week old male SHRs were purchased and housed in the Animal Facility at the Richardson Centre for Functional Foods and Nutraceuticals under a 12-h day and night cycle at 25 °C. After a 2-week acclimatization on regular chow, the SHRs were randomly divided into 4 groups of 8 rats each that received similar feed, but with the addition of hydrolyzed (HMH) or unhydrolyzed (HPI) hemp seed products to determine the oxidative stress-attenuating ability during the rapid growth phase (Trial I). The control diet contained 20% (w/w) casein, which was then substituted with 0.5% (w/w) HMH (19.5% casein + 0.5% HMH), 1% (w/w) HMH (19% casein + 1% HMH) or 1% (w/w) HPI (19% casein + 1% HPI). The diets were also formulated to contain same NaCl content, as previously reported [23]. After an 8-week *ad libitum* feeding period, 4 rats from each group were terminated and blood processed into plasma samples, which were then stored at −80 °C until needed for further analysis [23]. The remaining SHR were switched to regular chow diet for 4 weeks to serve as a washout period and allow the establishment of oxidative stress. After the washout period and to determine the potential treatment ability of HMH, the rats (now 20 weeks old) were randomized (4 each) to the four diet groups (20% casein; 0.5% HMH; 1% HMH; 1% HPI) followed by *ad libitum* feeding for 4 weeks (Trial II). In order to minimize any potential carry-over effect from Trial I, the rats were randomized, such that the mean systolic blood pressure (which has been shown to be directly related to oxidative stress in SHR [22]) was similar across the groups. At the end of the 4-week feeding period, all rats were terminated and blood collected to obtain plasma. The third feeding experiment (Trial III) used 20-week old NTRs, which were randomly assigned to 3 protein treatment groups with 6 rats per group: control diet (20%, w/w casein); 1% HMH diet (19% casein + 1% HMH); or 1% HPI diet (19% casein + 1% HPI). Only one HMH dose for the NTR study was used, because from the SHR experiments, the 1% HMH was a more effective dose than the 0.5% HMH in lowering plasma peroxide level. The NTR were allowed *ad libitum* access to their respective group feeds and tap water for 4 weeks, after which they were also terminated for blood collection and processing into plasma.

### 2.4. Plasma Total Peroxides (TPx) Assay

The plasma peroxide levels of rats were measured using a previously reported method [28], which was modified as follows. A 1-mL aliquot of rat plasma samples was mixed with 1.5 mL of 0.1 M sodium phosphate buffer, pH 7.0. For the blank assay, 1 mL of buffer was mixed with 1.5 mL of buffer. The sample mixtures and blank were warmed to 60 °C, and thereafter, 0.1 mL of each were mixed with 4.7 mL of 75% (v/v) ethanol in water solution, 0.1 mL of ammonium thiocyanate (30%, w/v) and 0.1 mL of 0.02 M ferrous chloride dissolved in 1 M HCl. An aliquot (0.2 mL) of this solution mixture was added to a clear bottom 96-well microplate, and the degree of color development was measured at 500 nm after 3 min of incubation at room temperature. The color intensity (absorbance value) is directly proportional to the peroxide level in the plasma sample.

### 2.5. Plasma Total Antioxidant Capacity (TAC) and Antioxidant Enzymes Assays

Plasma TAC, SOD and CAT activity were each analyzed using the respective OxiSelect assay kit (Cell Biolabs Inc., San Diego, CA, USA), according to the manufacturer’s instructions. For TAC, the samples were analyzed spectrophotometrically using a microplate reader. TAC values were calculated using measured absorbances at 490 nm and the results expressed as mM uric acid equivalents/mL of plasma. The SOD activity was determined using the xanthine/xanthine oxidase system to generate superoxide anions, which reduces a chromogen to produce a water-soluble formazan dye. The activity of SOD is determined as the inhibition of chromogen reduction. Briefly, the plasma (or buffer for the blank), xanthine solution, chromogen solution, 10 × SOD assay buffer and water were mixed and added into the clear 96-well microplate. Then, 10 μL pre-diluted xanthine oxidase solution was added into each well and the mixture incubated for 1 h at 37 °C. The absorbance was read at 490 nm on a microplate reader, and SOD activity was calculated based on the standard curve prepared with different SOD units as a function of the chromogen inhibition percentage as follows:

SOD activity (%) = (A_blank_ − A_sample_)/((A_blank_) × 100) (2)

The CAT assay was performed following the manufacturer’s instructions and reading the absorbance at 520 nm. CAT activity was calculated using a calibration curve and the results expressed as U/mL of plasma.

### 2.6. Statistical Analysis

Except where indicated, data were collected in triplicate and subjected to one-way analysis of variance using Statistical Analysis System Software (SAS version 9.2, SAS Institute, Cary, NC, USA). Significant differences were determined by Duncan’s multiple range test and accepted at *p* < 0.05.

## 3. Results

### 3.1. In Vitro Antioxidant Properties of HMH

**Figure 1:**
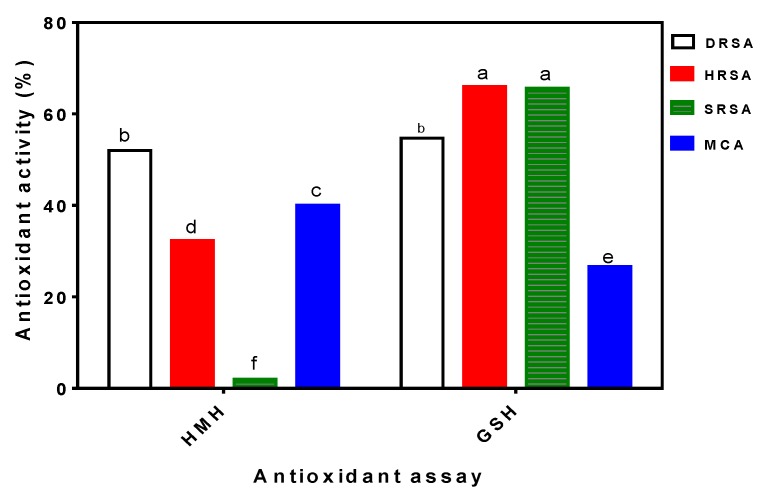
*In vitro* antioxidant properties of hemp seed meal hydrolysate (HMH) and glutathione (GSH) at 1 mg/mL concentration. DRSA, DPPH radical scavenging activity; HRSA, hydroxyl radical scavenging activity; SRSA, superoxide radical scavenging activity; MCA, metal chelating activity. Bars with different alphabets have mean values that are significantly different at *p* < 0.05. Values are means (*n* = 3) ± SD.

In order to estimate the potential antioxidant power prior to carrying out the rat feeding trials, HMH was evaluated at a 1-mg/mL protein concentration using various *in vitro* assays. Figure 1 shows that the HMH was able to scavenge up to 52% of the DPPH radicals in addition to scavenging the hydroxyl and superoxide radicals by 32 and 2%, respectively. The HMH also exhibited 40% MCA, which is an indication of a moderate capacity to form complexes with transition metals. Thus, the HMH could reduce the metal-catalyzed Haber-Weiss reaction and the accompanying formation of superoxide anions or other toxic hydroxyl radicals. The HMH has similar DPPH scavenging activity, but significantly (*p* < 0.05) lower hydroxyl radical scavenging activity (HRSA) and superoxide radical scavenging activity (SRSA) when compared to GSH. In contrast, Figure 1 shows that the HMH has significantly (*p* < 0.05) higher MCA than GSH.

### 3.2. Plasma SOD Activity

**Figure 2:**
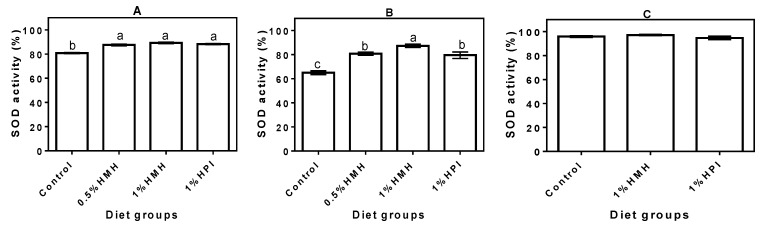
Superoxide dismutase (SOD) activity in plasma of (**A**) young growing spontaneously hypertensive rats (SHRs) (Trial I), (**B**) adult SHRs with established hypertension (Trial II) and (**C**) normotensive rats (Trial III) with a casein-only diet or casein diet that contained hemp seed meal protein hydrolysate (HMH) or hemp seed protein isolate (HPI). Bars with different letters have mean values that are significantly different (*p* < 0.05). Values are means (*n* = 4 rats) ± SD.

Figure 2 depicts the activities of SOD antioxidant enzyme in the plasma of SHRs (A and B) and NTRs (C) fed various levels of hemp seed peptides (HMH) and the isolate (HPI) in comparison to the control rats fed a casein only diet. The SOD activity in the growing SHRs that consumed hemp seed product-containing diets ranged from 88% ± 0.96% to 90% ± 1.29%, which was significantly (*p* < 0.05) higher when compared to 81% ± 0.96% activity in the control rats that consumed the casein-only diet (Figure 2A). SOD activity was reduced in all of the SHR groups during the adult stage, but that of the casein-only group (65% ± 3.27%) was still significantly (*p* < 0.05) lower when compared with the 80% ± 2.50% to 87% ± 5.45% values obtained for the hemp seed product-containing diets (Figure 2B). In contrast, the SOD levels in the NTR groups did not differ, but were higher (95% ± 1.73% to 98% ± 4.50%) than the levels observed in the plasma of all of the SHR groups (Figure 2C).

### 3.3. Plasma CAT Activity

**Figure 3:**
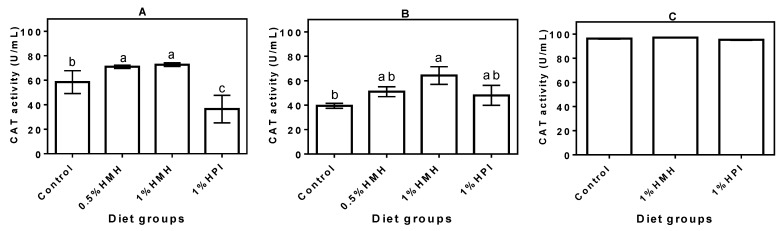
Catalase (CAT) activity in plasma of (**A**) young growing spontaneously hypertensive rats (SHRs) (Trial I), (**B**) adult SHRs with established hypertension (Trial II) and (**C**) normotensive rats (Trial III) with a casein-only diet or casein diet that contained hemp seed meal protein hydrolysate (HMH) or hemp seed protein isolate (HPI). Bars with different letters have mean values that are significantly different (*p* < 0.05). Values are means (*n* = 4 rats) ± SD.

In the growing phase, the SHR plasma CAT activity was significantly increased when hemp seed peptides (HMH), but not the unhydrolyzed protein (HPI), were part of the diet (Figure 3A). In fact, the HPI-containing diet had decreased the CAT level, which was significantly (*p* < 0.05) lower than that of the casein-only group. Plasma CAT activity decreased as the rats grew older, though the decrease was less for SHRs that consumed the hemp seed product-containing diets (Figure 3B). The CAT activity for the casein-only fed rats (Figure 3B) was significantly (*p* < 0.05) lower than that of the young rats fed the same diet (Figure 3A). In contrast, there were no significant differences when the CAT activities in the adult rats were compared with those of the young rats. SHRs that consumed the 1% HMH-containing diet had a significantly (*p* < 0.05) higher plasma CAT level when compared to those that consumed the casein-only diet. For the NTRs, there was no significant difference (*p* > 0.05) between the plasma CAT levels of the diet groups (Figure 3C); however, the NTRs had higher CAT levels when compared to the SHRs.

### 3.4. Plasma TAC

**Figure 4:**
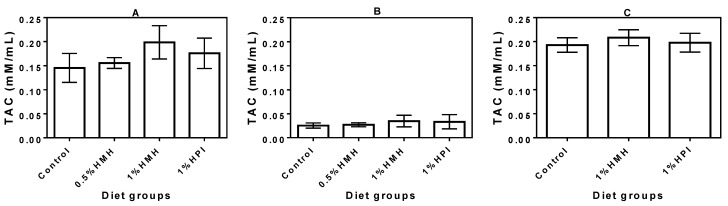
Total antioxidant capacity (TAC) in the plasma of (**A**) young growing spontaneously hypertensive rats (SHRs) (Trial I), (**B**) adult SHRs with established hypertension (Trial II) and (**C**) normotensive rats (trial III) with a casein-only diet or casein diet that contained hemp seed meal protein hydrolysate (HMH) or hemp seed protein isolate (HPI). There were no significant (*p* > 0.05) differences between the mean values within each plot. Values are means (*n* = 4 rats) ± SD.

The TAC in the plasma of growing SHRs that consumed hemp seed product-containing diets was slightly higher (especially for the 1% HMH diet) than the value obtained for the casein-only diet, though the differences were not significant (Figure 4A). Similar to the trend obtained for the SOD and CAT results, the adult SHRs showed significantly (*p* < 0.05) reduced TAC (Figure 4B) when compared to the growing SHR. The presence of hemp seed products in the diet had no beneficial effect on the SHR plasma TAC. The NTR showed slightly higher TAC levels (Figure 4C) when compared to the observed levels in growing or adult SHR, but there was also no diet effect.

**Figure 5:**
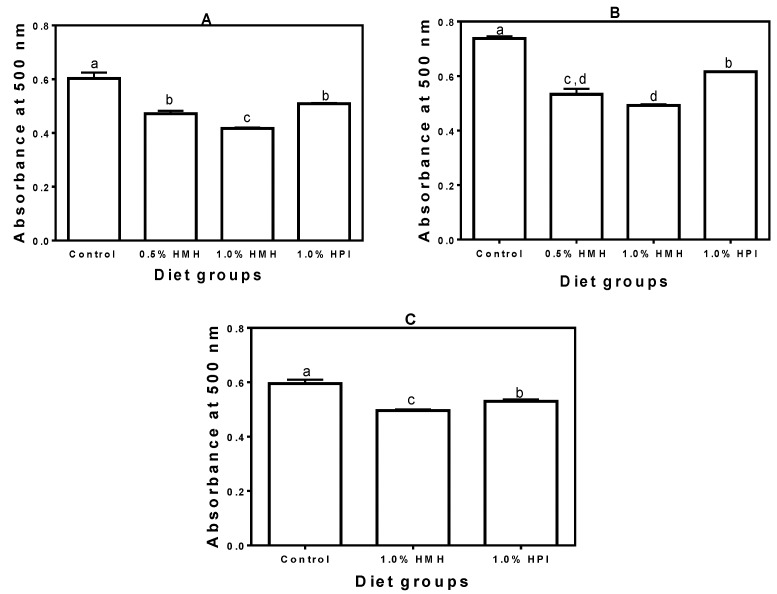
Total peroxide levels in plasma of (**A**) young growing spontaneously hypertensive rats (SHRs) (Trial I), (**B**) adult SHRs with established hypertension (Trial II) and (**C**) normotensive rats (Trial III) with a casein-only diet or casein diet that contained hemp seed meal protein hydrolysate (HMH) or hemp seed protein isolate (HPI). Bars with different letters have mean values that are significantly different (*p* < 0.05). Values are means (*n* = 4 rats) ± SD.

### 3.5. Plasma TPx

The growing SHR plasma TPx results showed a dose-dependent effect by the HMH in reducing lipid peroxidation, with the 1% inclusion level producing the lowest value, which was significantly (*p* < 0.05) better than the values obtained for the other diet groups (Figure 5A). The casein-only diet had a significantly (*p* < 0.05) higher plasma TPx level. In the adult SHRs, plasma TPx significantly (*p* < 0.05) increased by about 20% for the casein-only diet (Figure 5B) when compared to the growing SHRs. The 1% and 0.5% HMH diets produced significantly (*p* < 0.05) lower plasma TPx reduction than the casein-only and 1% HPI diets (Figure 5B). The 1% HMH diet also produced significantly (*p* < 0.05) lower plasma TPx in the NTRs followed by the HPI diet, while the casein-only diet had the highest value. Noticeably, the plasma TPx value for the casein-only fed NTRs (Figure 5C) was similar to that of the casein-only fed young SHRs (Figure 5A).

## 4. Discussion

The *in vitro* tests serve as a good predictive tool to test the potential antioxidative effects when the peptides are orally administered to animals. We used two broad types of antioxidant assays, the free radical scavenging ability (DPPH, hydroxyl and superoxide) and the MCA, to provide estimates of antioxidative effects. The free radical tests provide useful information on the hydrogen and electron-donating effects, while MCA provides information on the ability of the peptides to sequester reactive metal ions. Therefore, the results in Figure 1 show that the HMH contained peptides that can neutralize ROS in addition to preventing metal-catalyzed lipid peroxidation. Even though the DPPH radical is not a physiologically relevant species, its use gives an initial insight as to whether tested samples have the potential to be potent *in vivo* antioxidants against ROS and free radicals [29]*.* The results indicate that HMH contained peptides that are moderate scavengers of hydroxyl radicals, but poor scavengers of superoxide radical. The hydroxyl radical scavenging effect obtained for the HMH is very important because of the hyper-reactivity of this radical with almost every cellular molecule, including proteins, DNA, polyunsaturated fatty acids and cell membranes. These effects can cause severe damage to cells, produce a high oxidative stress condition and enhance the development or progression of chronic diseases, such as hypertension, cancer, diabetes and obesity [30]. The very low (2%) scavenging of superoxide radical by HMH is supported by our previous work [31], which showed that a hemp seed protein hydrolysate had no scavenging activity against the superoxide radical. The moderate MCA of the HMH indicates the potential to sequester ferrous and copper ions, which are agents that stimulate lipid peroxidation via the Fenton reaction. The MCA data also indicate the ability of the HMH peptides to reduce metal ion-catalyzed decomposition of hydroperoxides into reactive hydroxyl and alkoxyl radicals that perpetuate the damaging free radical chain reaction, because of their capacity to abstract hydrogen from surrounding biomolecules [27]. Since antioxidant mechanisms are diverse and employ different principles, the response of antioxidant ability determination may depend on various factors, such as the solvent and substrate used, affinity of substrate-antioxidant and the purity of the substrate, as well as that of the tested samples [32]. Therefore, this work supports a previous recommendation that antioxidant activity is better characterized using assays that are based on different mechanisms and using different media [21]. For example, if the HMH was tested for antioxidant activity using only the superoxide scavenging test, an inaccurate conclusion of inactivity may have been reached.

The feeding experiment data showed that oxidative stress was significantly (*p* < 0.05) higher in the adult SHRs that were fed the casein-only diet when compared to rats on diets that contained hemp seed protein products. Moreover, the reduced antioxidant defense (lower SOD, CAT and TAC) and higher TPx levels in the adult SHRs fed the casein-only diet are consistent with the fact that this rat model exhibits high oxidative stress during growth [22], which provides an excellent physiological environment to test antioxidant peptides. We have also recently reported that the HMH-containing diets attenuated hypertension development in the SHR through reductions in plasma levels of renin and angiotensin converting enzymes [23]. Therefore, our current report showing the antioxidant effect of the HMH-containing diets is consistent with the fact that high oxidative stress is an intrinsic physiological characteristic that contributes to hypertension development in the SHR. The anti-hypertension data [23] and current antioxidative results are consistent with data reported in a previous work that showed similar effects (systolic blood pressure and oxidative stress reductions) of an antioxidant drug (Tempol) after oral administration to SHRs [22]. Data from the feeding experiments confirm the *in vitro* results that showed the presence of antioxidative peptides within the HMH; these peptides were either absorbed into the blood or indirectly influenced the SHRs’ body homeostasis to enhance antioxidant defense capability. The antioxidant effect of the peptides was slightly lowered as the rats matured into hypertensive adults with the 0.5% and 1% HMH-fed SHRs exhibiting higher levels of SOD (80%–87%) in their plasma than that of the control (65%). The results are consistent with a previous report that suggested SOD activity in chronic hypertension may fall to below 60% if not treated, and age may be one of the contributing factors [33]. The higher SOD activity in the HMH-fed SHR groups could have contributed to lower plasma superoxide ion levels and, hence, the reduced TPx levels observed in their plasma when compared to the casein-only diet group. Our results also suggest that hemp seed peptides may have played a significant role in the enhancement of the CAT levels in plasma of treated SHRs, thus downregulating their oxidative stress status. CAT catalyzes the breakdown of H_2_O_2_ into water and O_2_ to reduce the amount of oxidative stress-promoting oxidants in circulation; however, this antioxidant defense mechanism becomes weaker during ageing or chronic fatigue and other disease conditions [34], which generally lead to reduced blood CAT levels. This trend is consistent with our data, which showed a significantly (*p* < 0.05) lower plasma CAT level (40%) in the adult SHR in comparison to the 58% in young SHRs when both groups were fed a casein-only diet. Therefore, the enhancement of CAT activity by the HMH peptides, especially in the adult rats, could also have contributed to the observed decrease in plasma TPx levels, which can decrease the risk or pathological intensity of cardiovascular disease. Previous studies [33,35,36] have also shown an age-associated increase in the concentrations of lipid peroxidation products. In contrast to the SHRs, the NTRs showed higher (95%–97%) plasma CAT activity in all of the diet groups, which is consistent with the lower oxidative stress level in the normal physiological state. Thus, the results confirm that CAT activity could be an important part of maintaining regular body homeostasis through increased elimination of H_2_O_2_ and associated toxic free radicals. The results on the major endogenous antioxidant enzymes are in agreement with previous reports that showed loach (*Misgurnus anguillicaudatus*) [37] and Sardinelle (*Sardinella aurita*) [38] protein hydrolysates reduced in *in vivo* oxidative stress through the enhanced activities of SOD and CAT.

TAC as a biological indicator of oxidative stress in biological fluids when measured in conjunction with individual antioxidant factors (such as SOD and CAT) or markers of oxidative damage could give a true picture of the oxidative status [39]. In this study, we have combined results of SOD, CAT and TAC to interpret the oxidative stress status of our model rats (SHR and NTR) fed hemp seed peptides in comparison to the control rats that consumed a casein-only diet. Generally, TAC was only slightly enhanced in the growing SHRs that consumed the 1% HMH-containing diet with a value of 0.199 ± 0.07 mM/mL when compared to the 0.145 ± 0.06 mM/mL value obtained for the casein-only diet. The TAC value for the SHRs on the 1% HMH diet is very similar to the 0.210 ± 0.05 mM/mL obtained for the 1% HMH diet-fed NTRs, which suggests the high antioxidant potential of the HMH peptides. The significantly (*p* < 0.05) low TAC values ((0.025 ± 0.008) – (0.035 ± 0.029) mM/mL) of the adult rats (Figure 4B) in comparison with the values ((0.145 ± 0.02) – (0.199 ± 0.07) mM/mL) observed for the young rats (Figure 4A) provides additional evidence of the rapid establishment of high oxidative stress during aging of the SHRs. Our data for the SHR are lower than the 1.22–1.44 mM/mL TAC values previously reported during the feeding of diets that contained frying oil [40]. Differences in TAC may be due to variations in diet composition. However, similar to our data, the frying oil study also showed that the NTRs had higher TAC values than the SHRs, which confirms the existence of a higher level of oxidative stress in the latter rat model. However, the TAC of the NTRs was not affected by the various diets used in this work.

Plasma peroxide levels are markers of the extent of lipid peroxidation at the cellular level. In biological systems, lipid peroxidation proceeds via a radical-mediated abstraction of hydrogen atoms from methylene carbons in polyunsaturated fatty acids, which initiates a sequence of reactions that generates aldehydes, ketones and other potentially toxic substances [41]. The HMH-containing diets produced the lowest peroxide levels, which suggest the ability of the peptides to reduce the rate of lipid peroxidation, either directly through metal chelation in the blood or indirectly through enhanced scavenging of free lipid peroxides. The results are similar to a previous report that used high potassium diets to reduce vascular and plasma lipid peroxide levels in stroke-prone SHRs [42]. The higher TPx level in the casein-only fed adult SHRs is consistent with the other results (lower CAT, SOD and TAC), which confirms reduced antioxidant capacity along with the accumulation of toxic free radicals as the rat aged. The fact that the HMH diets were more effective in reducing plasma TPx is an indication of higher bioactive potency of dietary peptides in comparison to whole proteins. The higher efficacy of peptides can be attributed to the faster rate of absorption and bioavailability, since there may be little or no need for additional digestion within the gastrointestinal tract (GIT). In contrast, the whole proteins present in HPI will require extensive digestion within the GIT, which could delay the transit of resultant bioactive peptides into the blood circulatory system. The higher potency of the HMH diets may also be due to the higher contents of aromatic amino acids (20.67%) and hydrophobic (29.13%) when compared to the HPI with 8.14% and 25.97%, respectively [23]. These amino acids enhance peptide interactions with lipophilic compounds, such as unsaturated fatty acids, and provide *in situ* electron or hydrogen donation, which contributes to faster quenching of free radicals in addition to downregulating the catalytic effects of lipid peroxidation-promoting transition metals. Unlike the other assays, the HMH diet led to reduced plasma peroxides in the NTRs, which could be an indication that some lipid peroxides are also produced during normal homeostasis. Thus, even under normal physiological conditions, consumption of HMH peptides may help prevent the accumulation of toxic lipid peroxides in the blood and reduce the risk of diseases associated with excessive levels of these radical compounds.

## 5. Conclusions

The *in vivo* evaluation of the antioxidant defense levels associated with dietary treatment of SHRs has shown that hemp seed protein and peptide products could be used to improve the oxidative stress status. The work also confirms that the SHR has an established high oxidative stress condition in the adult stage and serves as an excellent animal disease model to evaluate the bioactive potential of antioxidant compounds. Both the hydrolyzed (HMH) and unhydrolyzed (HPI) hemp seed protein products showed effective potential to reduce oxidative stress in the SHR, though the presence of pre-digested peptides probably contributed to the higher activity of the HMH. The results suggest that casein hydrolysis in the SHR GIT may have produced an inadequate level of antioxidant peptides or the peptides had poor potency after absorption, hence the inability of the casein-only diets to reduce oxidative stress. The action mechanism of the hemp seed peptides seems to involve the upregulation of antioxidant enzyme defense system in addition to radical scavenging and inhibition of lipid peroxidation. The SHR results correlate with the observed *in vitro* radical scavenging and metal chelating activities. Therefore, HMH may serve as an important ingredient to formulate antioxidant diets with potential therapeutic effects.
